# Otogenic Sigmoid Sinus Thrombosis in a 34-Year-Old Woman: A Rare Intracranial Complication

**DOI:** 10.7759/cureus.107094

**Published:** 2026-04-15

**Authors:** Yolanda F.Y. Lau, Ahmed Amer, Gerard Zed Ayaay, Alexander Schuster Bruce

**Affiliations:** 1 Emergency Medicine, Royal Surrey County Hospital, Guildford, GBR; 2 Radiology, Royal Surrey County Hospital, Guildford, GBR

**Keywords:** cerebral venous sinus thrombosis (cvst), intracranial complication, otitis media, otomastoiditis, sigmoid sinus thrombosis

## Abstract

Otogenic sigmoid sinus thrombosis (SST) is a rare but potentially life-threatening intracranial complication of otitis media, particularly uncommon in adults in the antibiotic era. Diagnosis may be delayed due to nonspecific early clinical features, with rapid deterioration secondary to raised intracranial pressure and impaired venous drainage. We report the case of a 34-year-old woman who presented to the emergency department with a four-day history of progressive right-sided otalgia, headache, and vomiting. She initially presented alert with a Glasgow Coma Scale (GCS) score of 15/15 but rapidly deteriorated to 5/15 (E2 V1 M2), requiring intensive care support. Neuroimaging revealed bilateral otomastoiditis with right tegmen tympani dehiscence and associated pneumocephalus, and further MRI brain revealed extensive non-occlusive thrombosis of the right transverse and sigmoid sinuses extending to the jugular bulb. Viral screen tested positive for respiratory syncytial virus (RSV), and blood cultures grew penicillin-resistant *Streptococcus pneumoniae*. The patient achieved complete sinus recanalisation and full neurological recovery with conservative management of targeted intravenous (IV) antimicrobial therapy, therapeutic anticoagulation, and neurocritical care, without surgical intervention. This case highlights that otogenic SST remains a medical emergency despite its rarity, and atypical clinical features should not diminish suspicion for severe intracranial pathology. Carefully selected patients without drainable collections or cholesteatoma may achieve excellent outcomes with prompt intensive medical management alone, supported by close radiological and neurological monitoring, even in the presence of extensive venous sinus thrombosis.

## Introduction

Intracranial complications of otomastoiditis, once common and frequently fatal, are now exceedingly rare in the antibiotic era [1]. Among these, lateral dural sinus thrombosis accounts for approximately 2-20% of all intracranial complications of otitis media, ranking third most common after meningitis and brain abscess [2]. Prior to the advent of antibiotics, the overall intracranial complication rate of suppurative otitis media was 2.3-4%, which has fallen to 0.04-0.15% thanks to modern antimicrobial therapy and advanced imaging and surgical techniques [3]. 

Within this group, otogenic sigmoid sinus thrombosis (SST) is a particularly rare subtype of lateral dural sinus thrombosis. Its precise incidence remains difficult to quantify due to its rarity. Gender distribution varies across case series, with one 15-patient cohort reporting 11 male and four female patients [4]. Cases are predominantly reported in children; adult cases are uncommon and more often associated with chronic ear disease rather than acute presentations [5].

Despite its rarity, otogenic SST remains a clinical emergency. Early symptoms are often nonspecific or may be masked by prior antibiotics, delaying diagnosis [6]. The classical “picket fence” pattern of spiking fevers due to septic emboli is now seldom seen [2]. More commonly, patients present with persistent otalgia, fever, and mastoid tenderness, sometimes accompanied by signs of raised intracranial pressure (ICP) such as severe headache and vomiting [2,7].

We report this case to highlight the risk of sudden and severe neurological decline in adult otogenic SST. Importantly, despite extensive transverse SST, the patient achieved complete recanalisation and full neurological recovery with anticoagulation and intensive medical therapy alone, without surgical intervention. This outcome supports a conservative approach for carefully selected patients under close multidisciplinary supervision.

## Case presentation

A 34-year-old woman presented to the Emergency Department with a sudden-onset severe headache rated 10/10 in intensity, associated with repeated vomiting over the preceding three hours. Of note, she reported a four-day antecedent history of progressively worsening right-sided otalgia and aural fullness. Her past medical history included obesity (body mass index (BMI) of 48.8 kg/m^2^), hypothyroidism, anxiety, depression, and dermatitis herpetiformis. Regular medications included thyroxine, sertraline, pregabalin, and propranolol. She had no known drug allergies.

On arrival, GCS was 15/15, although the patient was visibly distressed secondary to pain. She reported subjective reduction of hearing in the right ear but denied focal neurological symptoms, including limb weakness, visual disturbance, or diplopia. Observations were as follows: oxygen saturation 94% without supplementary oxygen, temperature 37.3°C, blood pressure 130/83 mmHg, heart rate 90 bpm, and respiratory rate 17 breaths per minute. Analgesia taken prior to attendance had provided minimal relief. 

Clinical examination revealed right mastoid tenderness without overlying erythema or swelling. Otoscopy demonstrated intact tympanic membranes bilaterally with no abnormality of the external auditory canals. A provisional diagnosis of acute otitis media was made, and the patient was commenced on IV fluids, single-dose IV paracetamol at 1 g, single-dose IV ondansetron at 4 mg, and single-dose IV amoxicillin at 500 mg as empirical antimicrobial therapy.

Six hours into her attendance, whilst under ongoing observation, the patient deteriorated markedly. She developed a fever of 38°C alongside progressive drowsiness and confusion, with her GCS fluctuating between 5/15 (E2 V1 M2) and 10/15 (E2 V3 M5). In view of her rapidly declining level of consciousness, she underwent endotracheal intubation.

**Figure 1:**
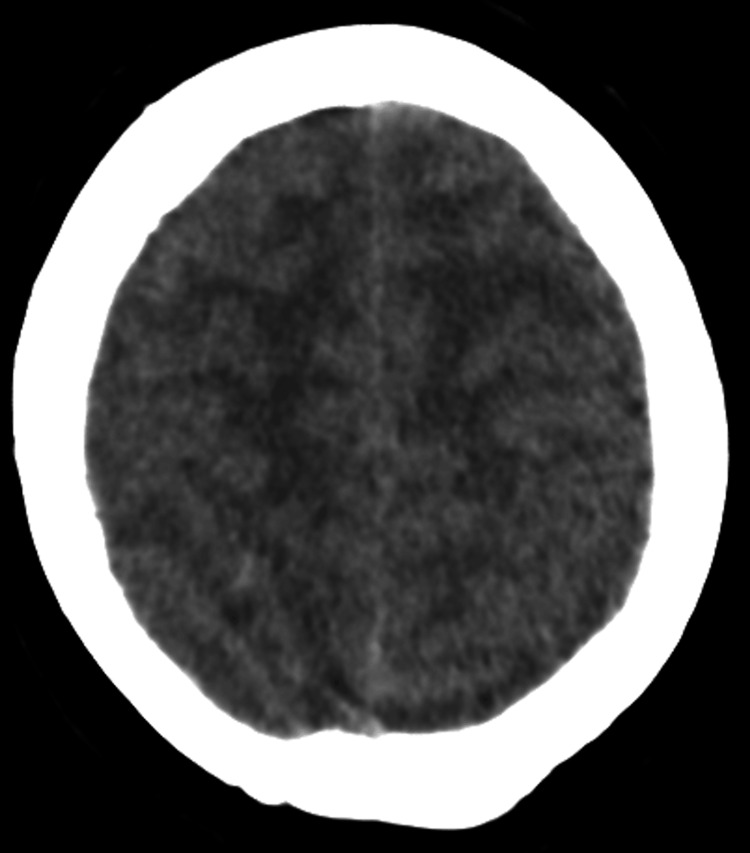
Non-contrast CT Head (axial view) demonstrating generalised sulcal effacement and obliteration of the ambient cisterns.

Respiratory viral screening returned positive for respiratory syncytial virus (RSV). Blood tests demonstrated a C-reactive protein (CRP) of 57 mg/L, white cell count 14.3 × 10*9/L with neutrophilia of 11.9 × 10*9/L, and monocyte count 1.0 × 10*9/L, consistent with active infection (Table 1). Non-contrast computed tomography (CT) scan of the head demonstrated diffuse cerebral swelling with generalised sulcal effacement and obliteration of the ambient cisterns, in keeping with cerebral oedema and raised ICP (Figure 1).

**Table 1:** Investigation results MIC: minimum inhibitory concentration

Blood Parameters	Reference Ranges	Patient Values
C-reactive protein	0.0-5.0 mg/L	57
Haemoglobin	115-165 g/L	146
Platelets	150-450 x 10*9/L	279
White blood cells	4.0-11.0 x 10*9/L	14.3
Neutrophils	2.0-7.5 x 10*9/L	11.9
Lymphocytes	1.0-4.0 x 10*9/L	1.2
Monocytes	0.2-0.8 x 10*9/L	1.0
Eosinophils	0.0-0.4 x 10*9/L	0.2
Basophils	0.0-0.1 x 10*9/L	0.1
Venous Blood Gas		
pH	7.32-7.43	7.45
Lactate	0.6-2.5 mmol/L	1.0
Blood cultures (aerobic and anaerobic)		
Strep pneumoniae (both bottles)	none	Positive
Resistant	-	Cotrimoxazole, erythromycin, penicillin (MIC =0.25), tetracycline
Susceptible	-	Levofloxacin, teicoplanin, ceftriaxone (MIC =0.38), linezolid

**Figure 2:**
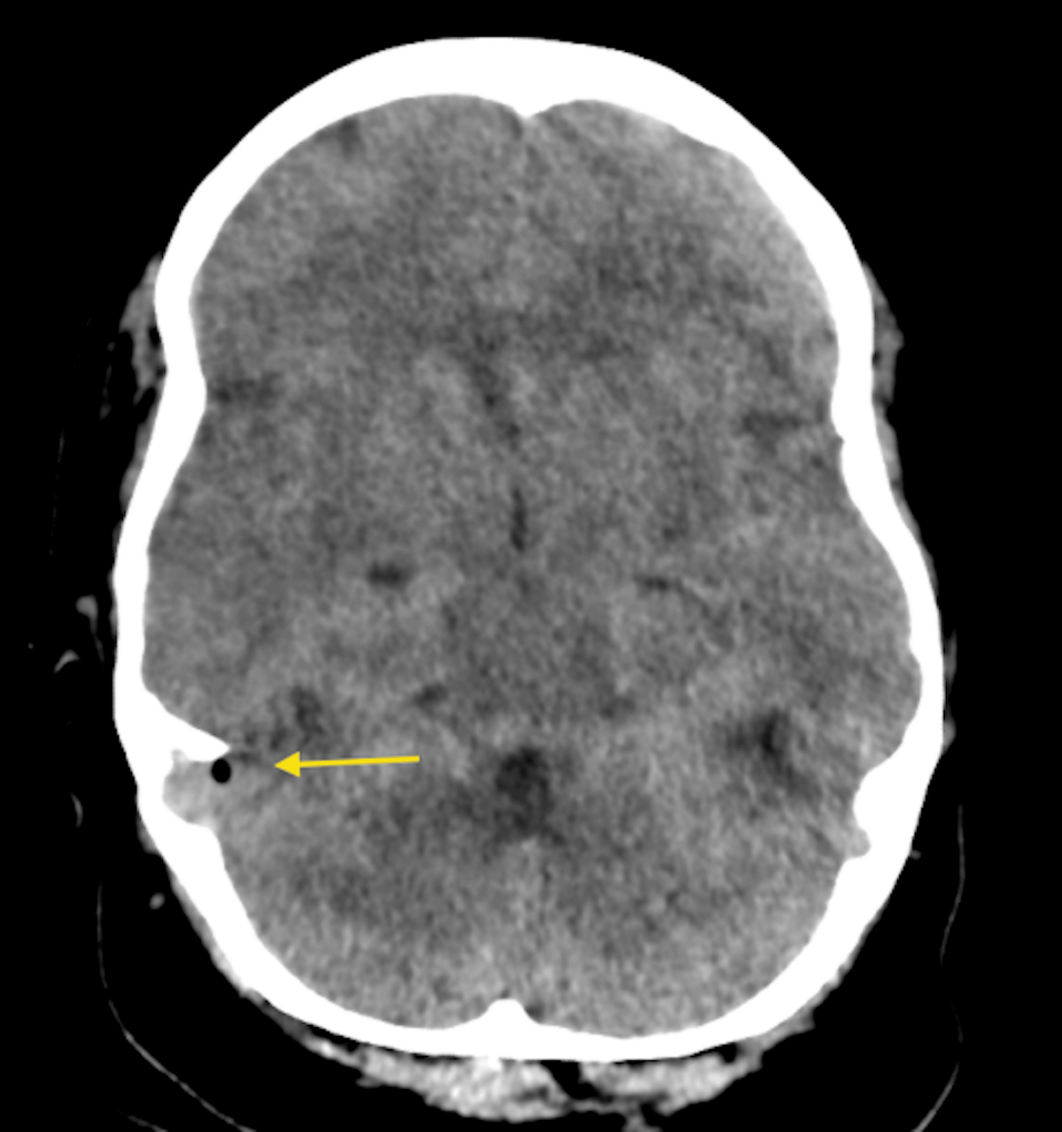
Non-contrast CT Head (axial view) demonstrating locules of intracranial gas consistent with pneumocephalus (arrow), located adjacent to the right sigmoid sinus.

Critically, abnormal gas locules were identified within the cranial cavity adjacent to the right sigmoid sinus, consistent with pneumocephalus, implicating a tegmen tympani dehiscence and establishing a pathological communication between the infected middle ear and the intracranial compartment (Figure 2). 

Following intubation, the patient was transferred to the ICU and commenced empirically on IV ceftriaxone at 2 g twice daily and weight-based IV aciclovir at 800 mg twice daily, alongside IV dexamethasone at 10 mg twice daily, given the strong clinical suspicion of pneumococcal intracranial infection. Ciprofloxacin 0.2% ear drops were prescribed bilaterally twice daily for local otological treatment. Neurocritical care measures included the use of moderate sedation and meticulous blood pressure control to maintain adequate cerebral perfusion pressure. Following radiological confirmation of dural venous sinus thrombosis (DVST), treatment-dose dalteparin at 12,500 units twice daily was initiated.

Blood cultures subsequently grew *Streptococcus pneumoniae*, confirming haematogenous dissemination of an invasive pneumococcal infection. Antimicrobial sensitivity profiling demonstrated penicillin resistance in the context of meningitis treatment (minimum inhibitory concentration (MIC) 0.25 mg/L), with susceptibility to ceftriaxone at the higher end of the susceptible range (MIC 0.38 mg/L) (Table 1). In light of these findings, IV linezolid at 600 mg twice daily was added to ensure adequate coverage of the resistant strain.

**Figure 3:**
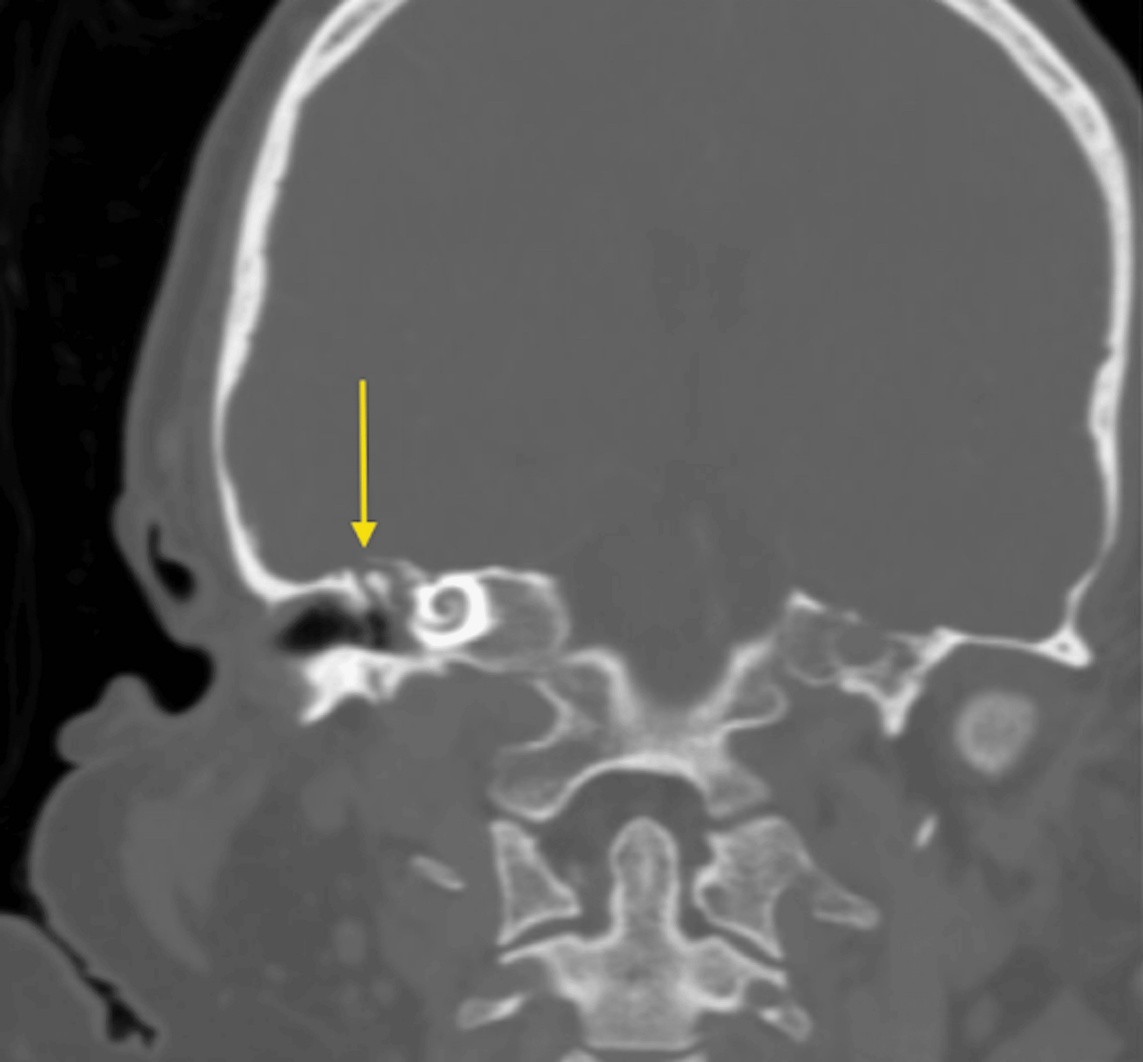
High-resolution CT Temporal Bones (coronal oblique reformat) demonstrates dehiscence of the right tegmen tympani (arrow) with loss of the bony plate separating the middle ear from the middle cranial fossa.

**Figure 4:**
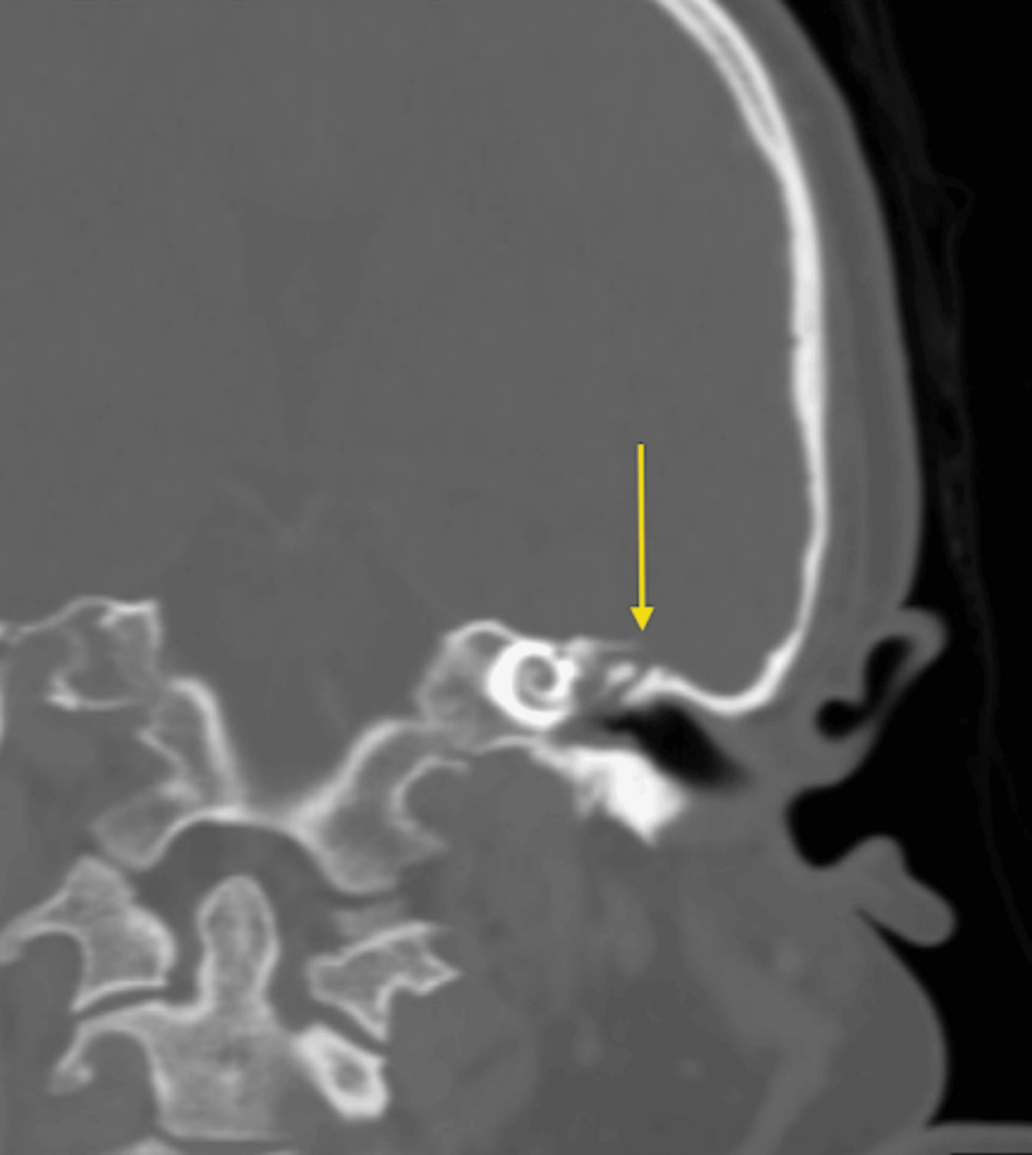
High-resolution CT Temporal Bones (coronal oblique reformat) demonstrates no definite dehiscence on the left tegmen tympani (arrow).

**Figure 5:**
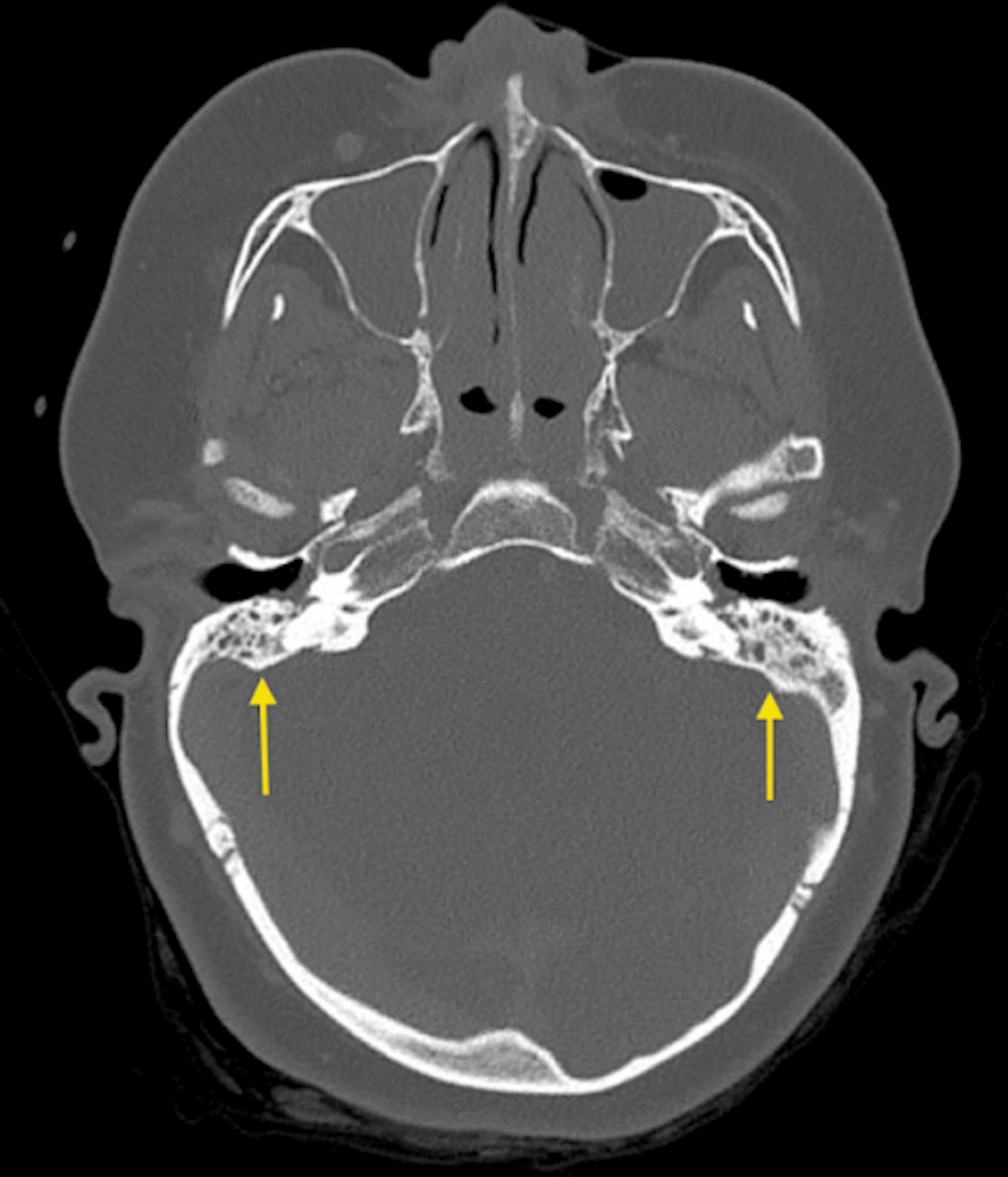
High-resolution CT Temporal Bones (axial reformat) demonstrates bilateral otomastoiditis with near-complete opacification of the mastoid air cells and middle ear cavities bilaterally (arrows). Hypoplastic and poorly pneumatised mastoid air cells were noted bilaterally, which may represent a congenital anatomical variant or changes secondary to previous otitis.

**Figure 6:**
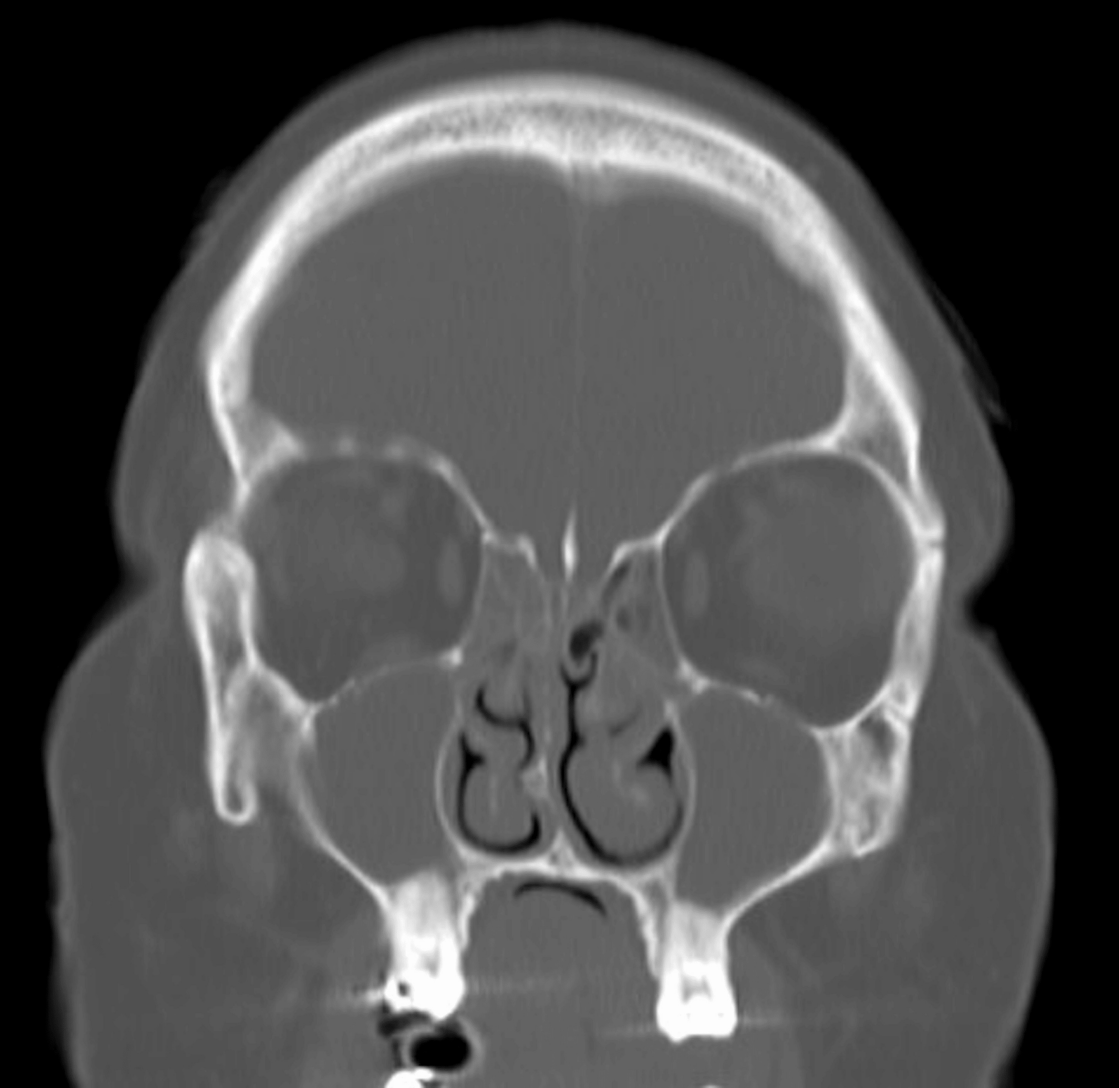
Contrast-enhanced CT Head with bone windows (coronal) demonstrates near-complete opacification of the bilateral maxillary sinuses and anterior ethmoid air cells.

Once haemodynamically stabilised, the patient underwent a dedicated high-resolution CT of the temporal bones and CT venography for further anatomical and vascular characterisation. Coronal oblique reformats confirmed a complete defect of the right tegmen tympani with erosion of the bony plate separating the right mastoid cavity from the middle cranial fossa (Figure 3), while no definite dehiscence on the left side (Figure 4). Axial reformat showed complete dehiscence of the right tegmen tympani with erosion of the bony plate separating the right middle ear and mastoid cavity from the middle cranial fossa. Opacification of the right mastoid air cells and middle ear cavity is consistent with acute otomastoiditis (Figure 5). A further contrast-enhanced CT head with bone windows showed near-complete opacification of the bilateral maxillary sinuses and anterior ethmoid air cells (Figure 6).

**Figure 7:**
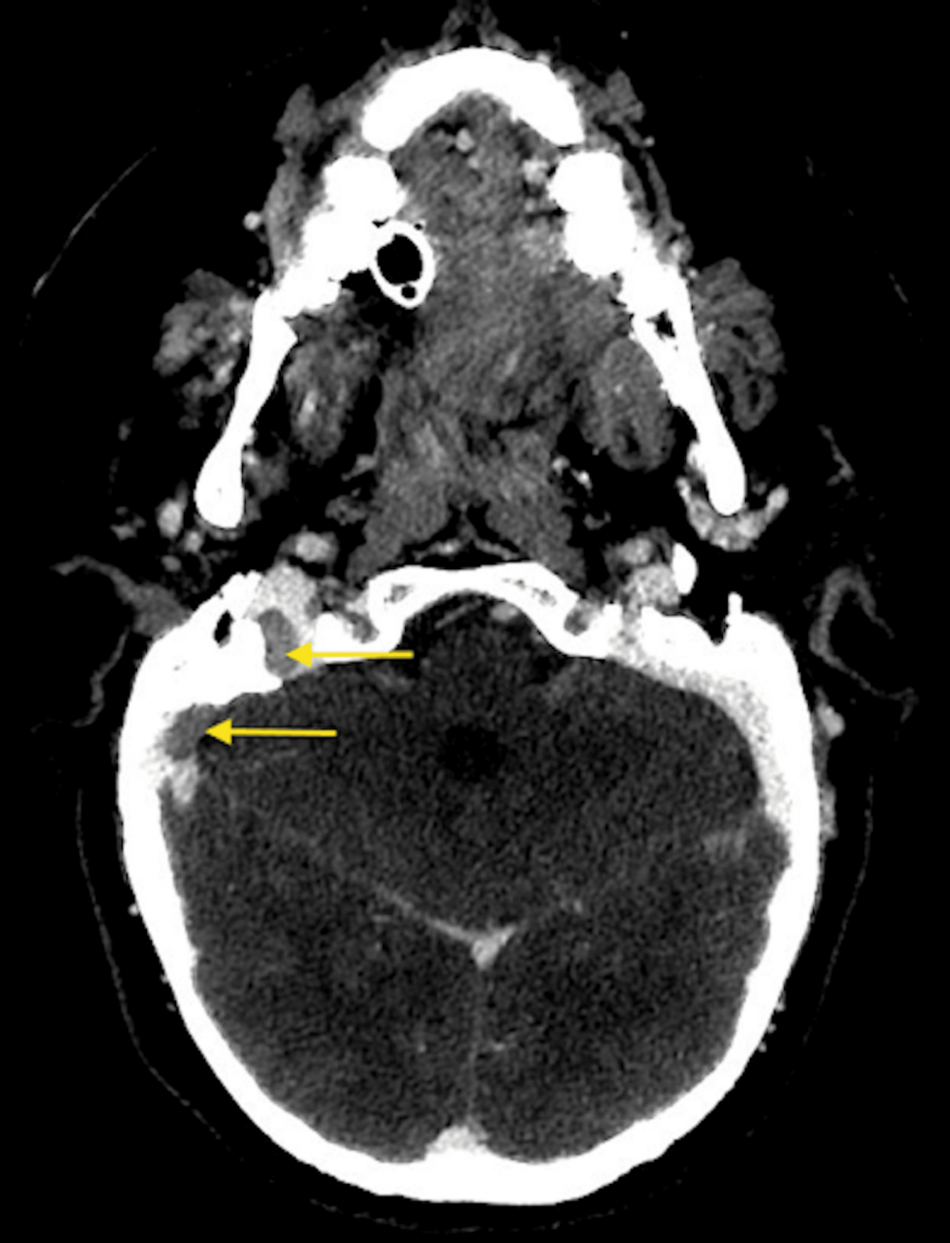
Cerebral CT Venography (axial) demonstrates an extensive filling defect within the distal right transverse sinus extending into the right sigmoid sinus and jugular bulb (arrows), consistent with non-occlusive DVST. DVST: dural venous sinus thrombosis

**Figure 8:**
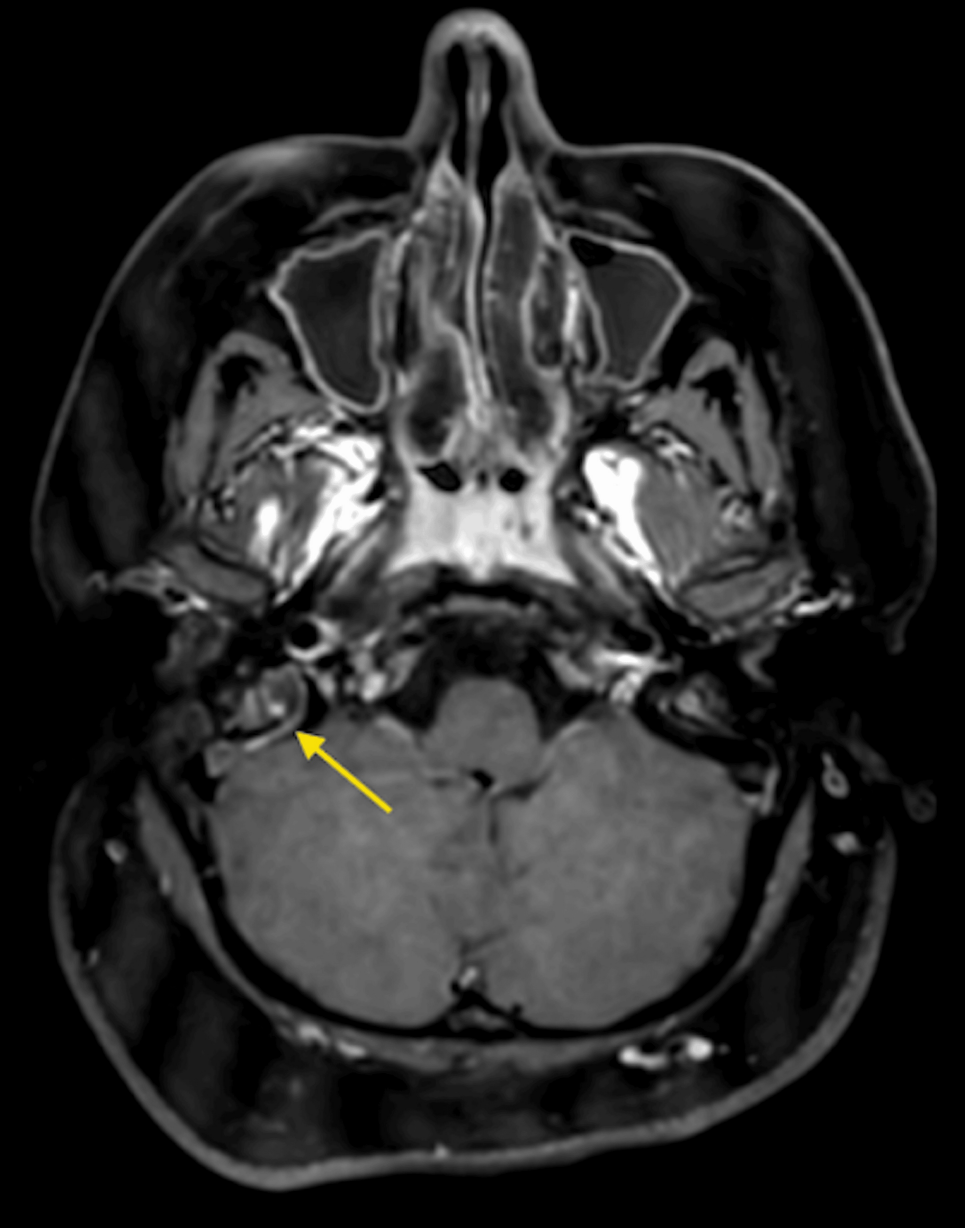
MRI Brain with gadolinium (T1, axial) shows abnormal hyperintense signal within the right distal transverse and sigmoid sinus (arrow) consistent with subacute thrombus.

CT venography initially demonstrated a non-occlusive thrombus in the distal right transverse sinus extending into the right sigmoid sinus and jugular bulb, with normal opacification of the left transverse and superior sagittal sinuses (Figure 7). This was corroborated by MRI brain demonstrating abnormal T1 hyperintense signal within the right sigmoid sinus in keeping with subacute thrombus. There was no evidence of parenchymal oedema, abnormal enhancement, or leptomeningeal enhancement (Figure 8). Diffusion-weighted imaging excluded acute ischaemic stroke, identifying only small foci of restricted diffusion confined to the thrombus within the right sigmoid sinus and a minor focus in the proximal left transverse sinus, effectively excluding acute parenchymal injury at this stage.

**Figure 9:**
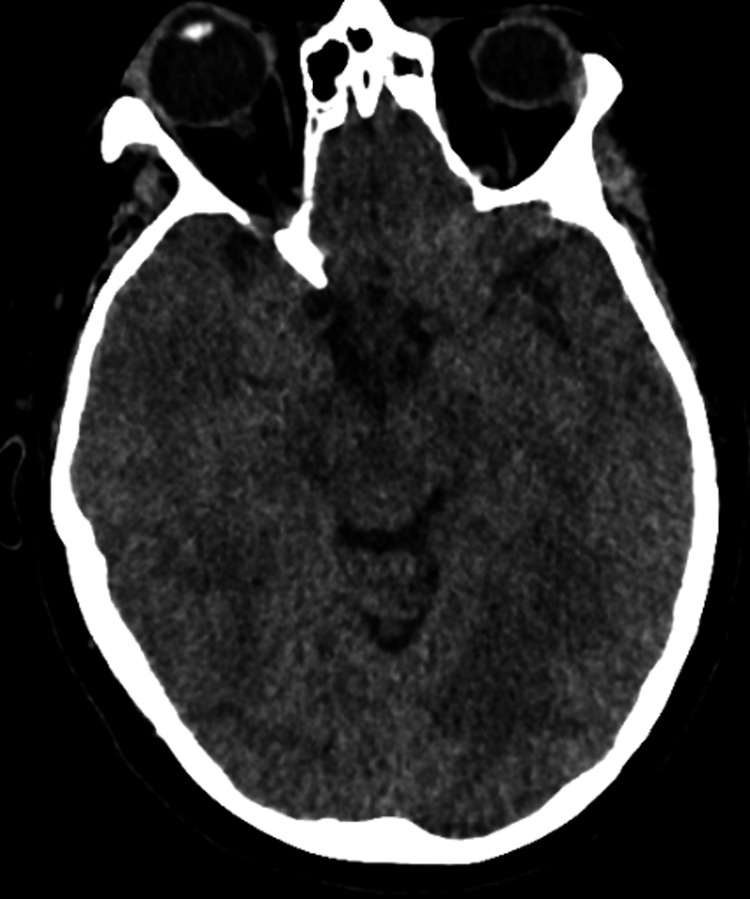
Urgent non-contrast CT Head (axial) obtained during the episode of hypertensive crisis and bradycardia, demonstrates marked effacement of extra-axial CSF spaces consistent with raised ICP. There is no evidence of transtentorial or tonsillar herniation, and basal cisterns remain patent.

On day four of her ICU admission, the patient developed bradycardia with a heart rate falling to 40-50 beats per minute alongside severe hypertension with a systolic blood pressure exceeding 190 mmHg, a constellation consistent with Cushing’s reflex and indicative of acutely raised ICP. Urgent non-contrast CT head confirmed marked effacement of the extra-axial cerebrospinal fluid (CSF) spaces in keeping with diffuse cerebral swelling, but excluded haemorrhage, new lesions, and transtentorial or tonsillar herniation, with the basal cisterns remaining patent (Figure 9). Raised ICP was managed with osmotherapy, maintenance of deep sedation, and blood pressure control. The episodes resolved over the subsequent 48 hours.

**Figure 10:**
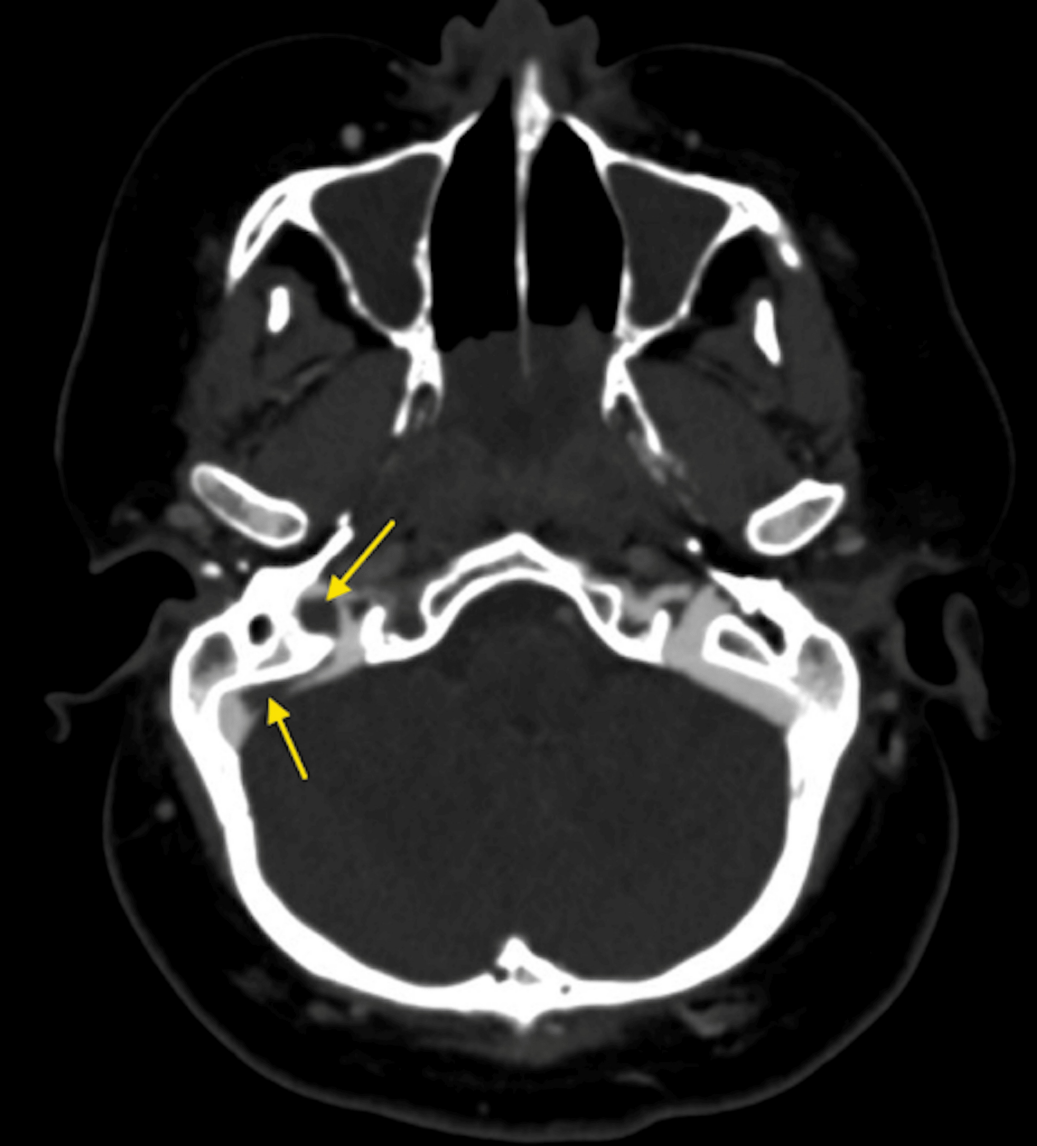
CT Head Venography (axial) demonstrates large non-occlusive thrombus in the right sigmoid sinus extending to the jugular bulb (arrows). No interval progression compared with baseline, indicating stable thrombosis under anticoagulation.

By day six, sufficient neurological improvement had occurred to allow cautious weaning of sedation for neurological assessment. Repeat CT venography demonstrated the right sigmoid sinus thrombus to be stable in extent with no progression to complete occlusion and no new thrombus formation (Figure 10).

The patient was successfully extubated on day 11 as her mental status normalised. IV ceftriaxone at 2 g twice daily and IV linezolid at 600 mg twice daily were continued for a total of 14 days, followed by a further week of oral antimicrobial therapy. Home medications were reinstated prior to discharge, and otological symptoms resolved progressively on serial examination.

**Figure 11:**
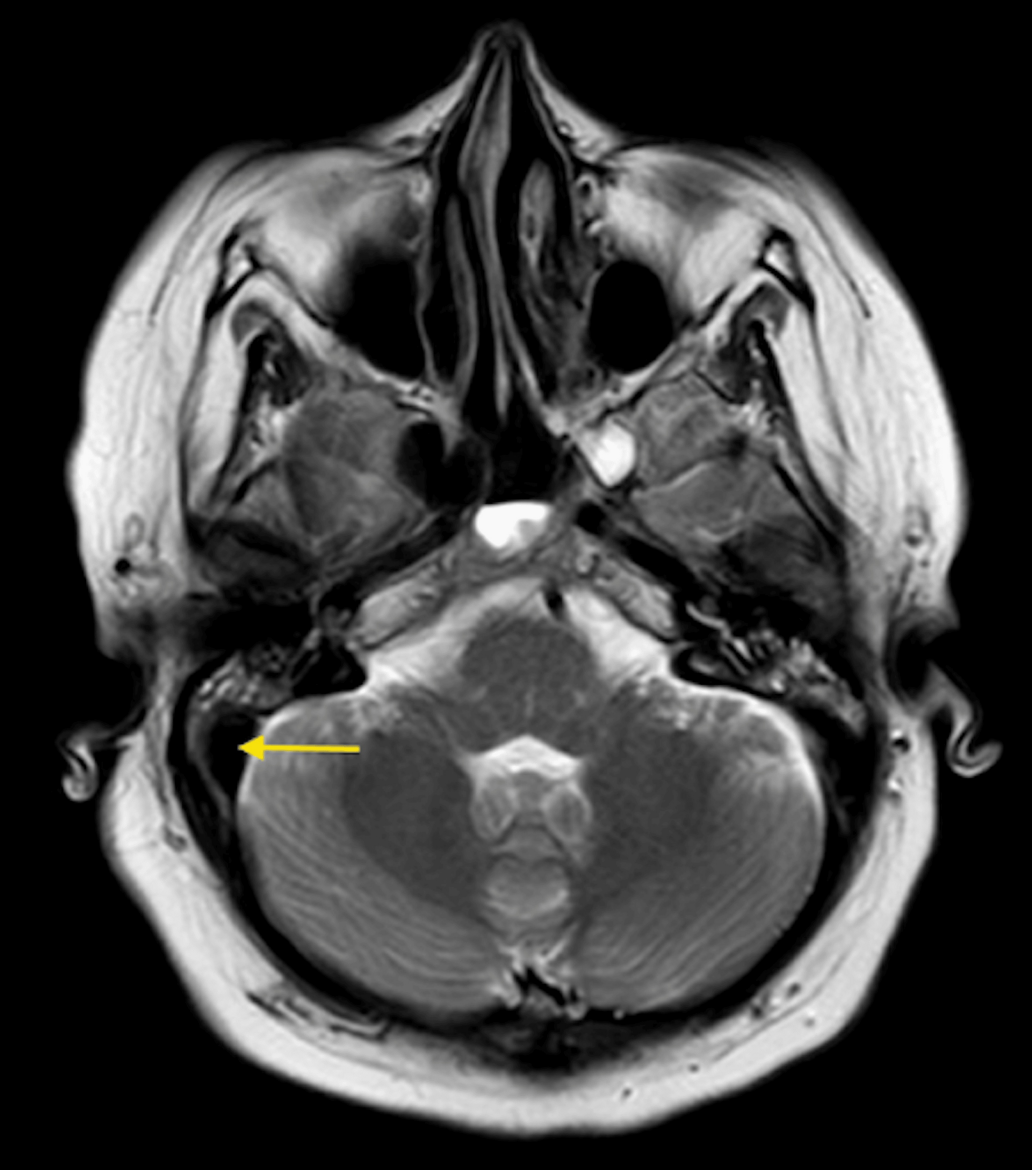
Follow-up MRI Brain with contrast on Day 21 (T2, axial) demonstrates signal void at site of previous thrombus, with no features of ongoing DVST. DVST: dural venous sinus thrombosis

Follow-up MRI brain obtained on day 21 demonstrated complete resolution of the DVST, with restoration of normal flow voids within the right sigmoid and transverse sinuses and no residual filling defects. Interval improvement in mastoid aeration indicated resolving otomastoiditis (Figure 11).

The patient was discharged home neurologically intact on a three-month course of anticoagulation with apixaban 10 mg twice daily for seven days followed by apixaban 5 mg twice daily, with interval imaging at three months to confirm sustained sinus recanalisation, and outpatient otolaryngology follow-up with planned formal audiological assessment following resolution of residual middle ear effusions.

## Discussion

Overview and pathophysiology

Otogenic SST represents one of the most severe intracranial complications of middle ear infection [4,8]. Although now rare in the antibiotic era, it continues to be associated with significant morbidity. Mortality rates for cerebral venous thrombosis (CVT) are reported at 5-10% generally [4], although more recent paediatric data suggest substantially improved outcomes, with no deaths reported in a systematic review of 312 cases of otogenic CVST [7].

From the pathophysiology perspective, infection spreads from the mastoid to the sigmoid sinus via two mechanisms: direct bony erosion by infectious granulation tissue or cholesteatoma, resulting in perisinus abscess formation that erodes through the sinus wall and endothelial damage; and thrombophlebitic propagation through small emissary veins from the infected area [5]. In both pathways, bacterial invasion and inflammatory mediators activate the coagulation cascade, leading to thrombus formation in the adjacent dural venous sinus [5]. Progressive propagation of the thrombus within the dural sinuses often extends from the sigmoid sinus into the transverse sinus and internal jugular vein [4]. As a result, occlusion of the sinus impairs venous drainage of the brain, leading to elevated ICP and its sequelae, potentially causing papilloedema, hydrocephalus, and cerebral oedema [5,6].

Clinical features, diagnosis, and microbiology 

This case presentation closely reflects the recognised features of otogenic SST in adults, including otological symptoms (otalgia with mastoid tenderness), systemic infection (leucocytosis), and raised ICP (severe headache, vomiting, and altered consciousness). Raja et al. reported headache, ear discharge, and hearing loss presented in all 15 patients in their study, with frequently associated nausea, vomiting, fever, and neck symptoms [4]. Similar patterns are seen in broader CVT cohorts, such as the 624 multinational adult cases from the International Study on Cerebral Vein and Dural Sinus Thrombosis (ISCVT), which reported headache occurrence of 88.9%, alongside seizures, focal neurological deficits, and altered mental status [8]. Collectively, these features reinforce the importance of early recognition and diagnostic vigilance when neurological symptoms complicate otomastoiditis. 

The microbiological findings in this case were notable for penicillin-resistant *S. pneumoniae* bacteraemia in the context of meningitis (MIC 0.25 mg/L), necessitating escalation from empirical amoxicillin to ceftriaxone with adjunctive linezolid. *S. pneumoniae* remains the most common organism in complicated acute otitis media with mastoiditis and intracranial extension, where *Streptococcus pyogenes* and *Staphylococcus aureus* are also frequently implicated [5]. Penicillin resistance is clinically significant due to its association with a more aggressive disease course and increased suppurative complications [9].

Respiratory viral screening was positive for RSV. While its precise contribution to the clinical course is uncertain, respiratory viruses are recognised as predisposing factors for acute otitis media [10]. Viral upper respiratory tract infection impairs eustachian tube function and decreases mucociliary clearance, facilitating nasopharyngeal bacteria ascension into the middle ear [11]. Viral co-infection, particularly *S. pneumoniae*, is known to increase the risk of suppurative ear disease [10].

An unusual feature of this case was the presence of bilateral mastoid involvement with unilateral thrombosis. Bilateral otogenic SST is extraordinarily rare [12], and the likely anatomical explanation relates to the right-sided tegmen dehiscence, which may have provided a direct route for intracranial extension of infection to the dural venous sinus [13,14].

The adult presentation of otogenic CVST also warrants emphasis. While complications of acute otitis media have traditionally been considered predominantly paediatric, contemporary series report an epidemiological shift toward adult patients, particularly with chronic ear disease [13,14]. Leskinen and Jero report a combined incidence of acute otological complications at 0.32 per 100,000 per year, with acute otitis media accounting for 80% of the underlying ear disease [14].

Diagnosis was confirmed by contrast-enhanced CT followed by CT venography, demonstrating bilateral coalescent mastoiditis with tegmen erosion and SST. In adults, multidetector CT angiography has shown excellent performance, with Linn et al. reporting 100% sensitivity and specificity in 19 patients, using MRI/MRV as the reference standard [15]. Raja et al. have similarly demonstrated 100% confirmation rate in 15 cases [4]. MRI with MRV remains the gold standard, offering a near 100% sensitivity and superior assessment of thrombus extent, early detection of venous flow abnormalities, and identification of associated complications such as meningeal enhancement, brain oedema, and septic emboli [11,15,16].

Management, outcomes, and risk factors

A key feature of this case was the patient's complete recovery without mastoidectomy or surgical intervention, despite radiological evidence of extensive otomastoiditis and documented transverse SST. Following discussion with a tertiary centre, conservative management was favoured as there was no cholesteatoma, drainable intracranial collection, or subperiosteal abscess to warrant mastoidectomy. The thrombus remained non-occlusive with preserved partial flow on serial CT venography, and the patient showed early clinical and radiological improvement after targeted antimicrobial therapy and therapeutic anticoagulation. Although mastoidectomy with or without sinus exploration has traditionally been used to eradicate the infective focus [4,6,8], contemporary evidence supports a conservative approach in carefully selected patients under close multidisciplinary surveillance [16,17]. 

The most debated aspect of otogenic SST management remains the role of anticoagulation [17,18]. The decision to anticoagulate this patient reflected careful assessment of risk versus benefit, informed by emerging systematic evidence supporting this approach [17]. George et al. (2023) in a landmark systematic review of 16 studies reported significantly higher rates of sinus recanalisation in anticoagulated compared to those not anticoagulated (76.67% versus 39.13%, p<0.001) [17]. Anticoagulated patients treated conservatively achieved a 93.75% recanalisation rate with a low complication rate of 12.50%, and a minimal rate of haemorrhagic complications attributable to anticoagulation at 1.77% [17]. Current guidelines therefore support anticoagulation for CVT for three to 12 months, depending on provoking factors and clinical response [11], with recanalisation most frequently occurring within the first three to six months [6,16]. 

The timing of anticoagulation required particular caution in this patient due to the raised ICP, pneumocephalus with tegmen dehiscence, and the theoretical risk of haemorrhagic transformation. Treatment-dose dalteparin was commenced after DVST confirmation, but before the episode of raised ICP on day four. Full therapeutic anticoagulation with heparin was introduced on day seven after stabilisation of ICP, and repeat imaging excluded haemorrhagic complications. This staged escalation may represent a pragmatic strategy in patients with competing risks, although evidence guiding optimal timing in the setting of raised ICP remains limited [11,17,18]. 

Intensive care management was central to the favourable outcome. Mechanical ventilation enabled neuroprotective strategies including controlled normocapnia, sedation, and head-of-bed elevation to optimise cerebral venous drainage [5,6]. The development of hypertensive bradycardia on day four raised concern for Cushing’s reflex, likely reflecting critically elevated ICP secondary to impaired venous outflow and venous congestion [5,6]. A single dose of IV dexamethasone was administered empirically for suspected pneumococcal meningitis but discontinued once the evolving clinical picture favoured primary DVST. Its role in isolated DVST without confirmed meningitis remains uncertain, and its use should be weighed carefully against the risk of immunosuppression in the context of active intracranial infection [11].

Formal thrombophilia screening in our patient was negative for inherited prothrombotic conditions, although several recognised acquired risk factors for CVT were present. Obesity (BMI >30) confers an approximately 2.63-fold increased risk of venous thromboembolism [19], and has also been associated with CVT, especially in women during reproductive years and in the presence of hormonal risk factors [8,19,20]. Local infection from otomastoiditis is a well-established precipitant of sigmoid and transverse sinus thrombosis [20]. In this case, the patient had at least three recognised risk factors: local infection, obesity, and female sex. 

This report is subject to the inherent limitations of a single case report. The favourable outcome achieved by conservative management cannot be generalised. In particular, the absence of cholesteatoma, non-occlusive thrombus, preserved venous flow, and early response to targeted antimicrobial therapy may not apply broadly. Furthermore, a substantial proportion of supporting evidence was derived from paediatric populations [4,5,6,16], so extrapolation to adult patients should be undertaken cautiously. Prospective multicentre studies are required to better define management parameters, including patients who can be managed safely without surgical intervention, and optimal timing and duration of anticoagulation.

## Conclusions

Otogenic SST is a rare but serious complication of otomastoiditis that still occurs even in the modern antibiotic era. This case highlights the need for a high index of suspicion in patients with ear infections who start developing severe headaches, neurological signs, or altered consciousness. Early imaging (CT venography) is crucial for diagnosis, and prompt multidisciplinary management can be life-saving. The effective treatment of otogenic SST hinges on eradicating the source of infection with antibiotics while managing the thrombotic process and ICP. With timely intervention, patients can achieve excellent outcomes with minimal residual deficits. This case serves as a reminder that clinicians must remain vigilant for intracranial complications of middle ear infections and be prepared to initiate intensive therapy when they arise.
